# Effect of LED Illumination Cycle and Carbon Sources on Biofilms of *Haematococcus pluvialis* in Pilot-Scale Angled Twin-Layer Porous Substrate Photobioreactors

**DOI:** 10.3390/bioengineering10050596

**Published:** 2023-05-16

**Authors:** Thanh-Tri Do, Toan-Em Quach-Van, Thanh-Cong Nguyen, Pau Loke Show, Tran Minh-Ly Nguyen, Duc-Hoan Huynh, Dai-Long Tran, Michael Melkonian, Hoang-Dung Tran

**Affiliations:** 1 Faculty of Biology-Biotechnology, University of Science, Vietnam National University Ho Chi Minh City, 227 Nguyen Van Cu Street, District 5, Ho Chi Minh City 72711, Vietnam; tridt@hcmue.edu.vn; 2 Faculty of Biology, Ho Chi Minh City University of Education, 280 An Duong Vuong Street, District 5, Ho Chi Minh City 72711, Vietnam; 3 Faculty of Biotechnology, Nguyen-Tat-Thanh University, 298A-300A Nguyen-Tat-Thanh Street, District 4, Ho Chi Minh City 72812, Vietnam; 4 Department of Chemical Engineering, Khalifa University, Abu Dhabi P.O. Box 127788, United Arab Emirates; showpauloke@gmail.com; 5 Faculty of Business Administration, TU Bergakademie Freiberg, Akademiestraße 6, 09599 Freiberg, Germany; minh-ly.nguyen-tran@student.tu-freiberg.de; 6 Can Gio Protection Forest Management Board, 1541 Rung Sat Street, An Thoi Dong Commune, Can Gio District, Ho Chi Minh City 73311, Vietnam; 7 Department of Supervisor Inspector, Van Lang University, Nguyen Khac Nhu Street, Co Giang Ward, District 1, Ho Chi Minh City 71013, Vietnam; 8 Department of Plant-Microbe Interactions, Integrative Bioinformatics, Max Planck Institute for Plant Breeding Research, 50829 Cologne, Germany; 9 Central Collection of Algal Cultures (CCAC), Faculty of Biology, University of Duisburg-Essen, 45117 Essen, Germany; 10 Faculty of Biology and Environment, Ho Chi Minh City University of Food Industry, 140 Le Trong Tan Street, Tay Thanh Ward, Tan Phu District, Ho Chi Minh City 72009, Vietnam

**Keywords:** astaxanthin, biofilm, *Haematococcus pluvialis*, LED, porous substrate photobioreactor, sources of carbon

## Abstract

Light-emitting diodes are increasingly used as artificial light sources in *Haematococcus pluvialis* cultivation due to the fact of their energy advantages. The immobilized cultivation of *H. pluvialis* in pilot-scale angled twin-layer porous substrate photobioreactors (TL-PSBRs) was initially performed with a 14/10 h light/dark cycle and showed relatively low biomass growth and astaxanthin accumulation. In this study, the illumination time with red and blue LEDs at a light intensity of 120 µmol photons m^−2^ s^−1^ was increased to 16–24 h per day. With a light/dark cycle of 22/2 h, the biomass productivity of the algae was 7.5 g m^−2^ day^−1^, 2.4 times higher than in the 14/10 h cycle. The percentage of astaxanthin in the dry biomass was 2%, and the total amount of astaxanthin was 1.7 g m^−2^. Along with the increase in light duration, adding 10 or 20 mM NaHCO_3_ to the BG11-H culture medium over ten days of cultivation in angled TL-PSBRs did not increase the total amount of astaxanthin compared with only CO_2_ addition at a flow rate of 3.6 mg min^−1^ to the culture medium. Adding NaHCO_3_ with a 30–80 mM concentration inhibited algal growth and astaxanthin accumulation. However, adding 10–40 mM NaHCO_3_ caused algal cells to accumulate astaxanthin at a high percentage in dry weight after the first four days in TL-PSBRs.

## 1. Introduction

In microalgal biotechnology, the porous substrate photobioreactor (PSBR) has been discussed as an emerging design [1,2,3]. Many tools for screening the optimal conditions of algal growth have been investigated in PSBRs. The cultivation of *H. pluvialis* to obtain astaxanthin has been studied and developed in a twin-layer porous substrate photobioreactor (TL-PSBR). Immobilized algae grown in TL-PSBRs can overcome some of the disadvantages of suspension systems [1,4,5]. Both the green and red phases of *H. pluvialis* can be cultured simultaneously in the same TL-PSBR system, with a shortened cultivation time [6]. The renewal or addition of nutrient medium during the culture period in the system can also be conducted easily, because the medium is always separated from the algal biomass. The harvesting step by scraping the microalgae biofilm is also performed with less energy input than in suspended cultivation [1,2,3]. However, knowledge concerning the influence of environmental factors on the growth and astaxanthin accumulation of *H. pluvialis* in suspension systems may not be appropriate when applied to TL-PSBRs [1,4,5]. A few small-scale laboratory studies on culturing *H. pluvialis* in TL-PSBR have investigated the effects of light intensity, light source, and CO_2_ addition in the gas phase [6,7,8]. Light is one of the most influential factors in the green stage and the encystment process of *H. pluvialis* [9,10,11]. Large-scale angled TL-PSBRs have also been established successfully and used in an experiment investigating the illumination efficiency of two different sodium luminaires [12]. However, due to the problem of energy consumption and heat generation, the sodium lamps in the angled TL-PSBRs were gradually replaced by LEDs in recent experiments [13,14]. Studies in suspension culture systems show that the light cycle also significantly affects astaxanthin production [9,15,16]. In our initial study, immobilized cultivation in angled TL-PSBRs with red and blue LEDs with a light/dark cycle of 14/10 h yielded a relatively low total dry biomass, 40.74 g m^−2^ [13]. In artificial light cultivation systems, LEDs may be suitable for increased light duration compared to sodium lamps due to the fact of temperature control issues [6,8,13]. However, the optimal light–dark cycle for the growth and accumulation of astaxanthin by *H. pluvialis* in TL-PSBRs must be determined.

The carbon source is also a factor that significantly affects growth and astaxanthin content in *H. pluvialis*. In suspended cultivation systems, algal cells can only utilize carbon dissolved in the culture medium. In TL-PSBR systems, the algae are close to CO_2_ in the gas phase on the surface of the biofilm, thus reducing the diffusion path of dissolved CO_2_ significantly [5,17]. Kiperstok et al. showed that adding 5% CO_2_ (*v*/*v*) to the air on the biofilm surface in a small-scale, vertical TL-PSBR system resulted in high growth efficiency [6]. However, adding CO_2_ to the air in large-scale TL-PSBRs with ventilation to regulate temperature and humidity is not feasible. Therefore, the optimization studies of carbon addition in a pilot-scale TL-PSBR system should be focused on selecting the appropriate carbon source and concentration, when directly added to the nutrient medium [5]. The primary sources of inorganic carbon commonly used are CO_2_ and bicarbonate (NaHCO_3_/Na_2_CO_3_) [18,19,20]. Each carbon source has advantages and disadvantages and different effects on the growth and accumulation of astaxanthin [21]. Therefore, reasonable control of carbon supplementation is also an issue that should be considered in astaxanthin production from *H. pluvialis* [5].

In this study, increasing the illumination time with combined red and blue LEDs was performed to increase the biomass and stimulate the astaxanthin accumulation of *H. pluvialis* in pilot-scale angled TL-PSBRs. In addition, the source and concentration of inorganic carbon added to the *H. pluvialis* culture medium in the angled TL-PSBR were also determined.

## 2. Materials and Methods

### 2.1. H. pluvialis Strain and Suspended Cultivation for Initial Biomass of Biofilm

The microalgal strain *H. pluvialis* CCAC 0125 was obtained from the Central Collection of Algal Cultures (https://www.uni-due.de/biology/ccac/) accessed on 1 January 2020. and kept on agar plates at 20 °C. To initiate experiments in the angled TL-PSBR, algae were transferred into liquid culture in 100 mL, 500 mL, and 2 L flasks at 26 ± 2 °C and illuminated by fluorescent lamps with the light intensity of 40–60 µmol photons m^−2^ s^−1^.

### 2.2. Experimental Set-Up

The experiments were performed in pilot-scale angled TL-PSBRs (0.5 m^2^ × 4) according to the description of Tran et al. [8]. A separate set of LEDs illuminated each chamber (0.5 m^2^) of the TL-PSBRs. Each set of LEDs consisted of 16 Bridgelux LED chips (Synergy Electronics (Shenzhen) Co., Ltd., Shenzhen, China). The LED chips used were red and blue with a ratio of 3:1 [13]. The LEDs were installed in a steel frame and controlled to turn on and off automatically using a timer. Light intensity was measured with a PG100N spectral PAR meter (UPRtek, Taiwan). The distance from the LEDs to the biofilm was adjusted to ensure that the light intensity on the algal surface was 120 µmol photons m^−2^ s^−1^.

#### 2.2.1. Experiment on the Effect of the Light/Dark Cycle of Blue/Red LEDs on the Growth and Astaxanthin Accumulation of *H. pluvialis* in Angled TL-PSBRs

The angled TL-PSBR was an open system, so this experiment aimed to maximize the biomass and astaxanthin content obtained in the shortest time to limit contamination. Therefore, the experiment of increasing the duration of the light regime in each light/dark cycle was conducted to promote photosynthesis of *H. pluvialis* during a short cultivation period (10 days). The light/dark cycle experiment included six treatments: 14/10 (control), 16/8, 18/6, 20/4, 22/2, and 24/0 h (continuous light). The light/dark cycle experiments were performed in different chambers.

Algae samples in the plots were collected on days 4, 6, 8, and 10 to determine the total dry biomass and astaxanthin content. The initial density of the algal biomass was 7.5 g m^−2^ [12]. In each chamber, algae were immobilized as circular biofilms with a radius of 5 cm to facilitate the sample collection. In this experiment, the pH value was maintained in a range of 6.5 to 8 by aerating fresh air with a 1% CO_2_ supplement (*v*/*v*) into the BG11-H medium [12].

#### 2.2.2. Experiments on the Effect of Carbon Source Supplementation on Growth and Astaxanthin Accumulation of *H. pluvialis* in Angled TL-PSBRs

In these experiments, algae were immobilized and grown for ten days at 24–26 °C with a light/dark cycle of 22/2 h and a light intensity of approximately 120 µmol photons m^−2^ s^−1^ from red and blue LEDs.

Effect of the amount of CO_2_ added to the medium on *H. pluvialis* in angled TL-PSBRs: In this experiment, CO_2_ was added to the BG11-H medium by a system of pressure-regulating valves and ceramic CO_2_ effervescent tubes to create small air bubbles, increasing the solubility of CO_2_ into the solution. The more dissolved CO_2_, the lower the pH of the environment. The growth of *H. pluvialis* is most favorable at a pH in the range of 6.5–8 [22,23], so we applied, on the angled TL-PSBR, the amount of CO_2_ while still ensuring that the pH remained within a favorable range. On this basis, the experiment was conducted including the following treatments: aeration of natural air (without CO_2_ supplement) into the culture medium (control) and aeration of pure CO_2_ into the BG11-H medium in the amounts of 3.6, 5.4, 7.2, and 9.0 mg min^−1^ (corresponding to a volume of 2, 3, 4, and 5 mL min^−1^).

Effect of the concentration of NaHCO_3_ dissolved in the medium on *H. pluvialis* in angled TL-PSBRs: Previous studies on the effect of bicarbonate ions on the growth and astaxanthin accumulation of *H. pluvialis* were mainly performed in suspension culture systems [22,23]. However, these studies also formed the basis for choosing NaHCO_3_ concentrations for experiments in the angled TL-PSBRs. The experiment to study the effect of NaHCO_3_ supplemented in BG11-H medium on the growth of the *H. pluvialis* biofilm and accumulation of astaxanthin included concentrations of 0 mM, 10, 20, 30, 40, 50, 60, 70, and 80 mM. Each experiment was conducted in a chamber with a medium supply system. The medium solution was also supplemented with CO_2_ gas in the amount of 3.6 mg min^−1^ to provide a carbon source along with NaHCO_3_. Due to the basicity, the addition of only NaHCO_3_ causes the pH of BG11-H medium to increase to higher than 8 and may affect the results of this experiment. Therefore, the addition of CO_2_ is necessary to ensure that the environmental pH is always in the range of 6.5–8, which is favorable for *H. pluvialis* growth. The criteria for comparison and evaluation were dry biomass productivity, the percentage of astaxanthin in the dry biomass, and the accumulation of astaxanthin in a m^−2^ growth area.

### 2.3. Methods

#### 2.3.1. Immobilization of Concentrated Algal Suspension and Cultivation in the Pilot-Scale Angled TL-PSBRs

The green phase of *H. pluvialis* CCAC 0125 was cultured in a suspension in 2 L flasks containing 1.5 L of BG11-H medium. After 16–18 days, the algal cells were centrifuged for 5 min at 800× *g*. The supernatant was removed, and the pellet was collected. On average, each liter of suspension had a final density of 1.2 g of dry algal biomass. Therefore, it was necessary to harvest approximately 6.25 L of the algal suspension to collect enough biomass for creation of the inoculum for 1 m^2^ of growth area in the TL-PSBR (7.5 g m^−2^). After centrifugation, the concentrated algal suspension was adjusted to a density of 0.1 g mL^−1^, so the volume of the inoculum for a 50.24 cm^2^ biofilm was 0.38 mL. The suspension was painted with a soft brush in circular plots with a radius of 4 cm (50.24 cm^2^ for each plot).

Cultivation of algae in the biofilms after immobilization in the angled TL-PSBRs: The time allowed for biofilm cultivation was ten days. The BG11-H medium with replete nutrients was used from day 1 to 7. The medium in the tank was then replaced with N- and P-deficient BG11-H from day 8 to 10 to induce nutritional stress on *H. pluvialis* cells. The pH of the medium was adjusted to 6.5–8, but it depended on the amount of carbon source supplemented into the medium in the experiments. The temperature was set to 24–26 °C.

#### 2.3.2. Analytical Methods

Sampling and determination of dry biomass of microalgae in the biofilm: Algal biofilm samples were collected to determine the total dry biomass on days 4, 6, 8, and 10 after immobilization. The substrate’s biomass in a circular plot (r = 4 cm) was scraped with a plastic pad and placed in pre-weighed paper bags (m_1_). Then, the algae were dried at 105 °C for two hrs, cooled, and weighed. The drying process was repeated until the weight was constant, and the total weight of the bag and the dried algal biomass (m_2_) were obtained. The following formula calculates the dry algal biomass per square meter: m (g m^−2^) = (m_2_ − m_1_)/πr^2^.

Determination of the percentage of astaxanthin in the dry algal biomass: The dried algal biomass was mixed well before being weighed to take 0.001 g into a 2 mL centrifuge tube. Then, 0.5 mL of 90% acetone and a stainless-steel ball (r = 2.5 mm) were added. The mixture was shaken on a shaker with a shaking amplitude of 20 mm and shaking frequency of 220 rpm to disrupt algal cells for two hrs. The sample was centrifuged at 1000× *g* for 2 min to collect the supernatant. Then, 0.5 mL of 90% (*v*/*v*) acetone was added to the residue, and the procedure was repeated until the supernatant obtained was colorless. Acetone (90%) was added to the extract to obtain a final volume of 2 mL in a centrifuge tube with a cap. The procedure was performed in the dark or low-intensity diffused light to avoid astaxanthin degradation. The pigment extracts were spectrophotometrically measured at 530 nm [6]. The concentration of astaxanthin was determined based on the standard curve equation established previously: x = (y − 0.0481)/0.0531, where y is the OD value, and x is the astaxanthin concentration (µg mL^−1^) [12]. The following formula calculates the percentage of astaxanthin in the dry biomass: A(%) = (x × 2) × 100%/1000.

Observation of cell morphology. Algal cells in the biofilm were sampled, suspended in fresh culture medium, fixed with 2% formol, and morphologically observed with an optical microscope by Carl Zeiss Primo Star (Germany).

### 2.4. Statistical Analysis and Graphing

Tukey’s HSD test was used for statistical analyses of the experimental results. The graphs were plotted with the R language (version 3.5.2). The values in the graph include the mean ± standard deviation (SD) of at least three biological replicates (n ≥ 3).

## 3. Results

### 3.1. Effect of the Light/Dark Cycle of Blue/Red LEDs on Growth and Astaxanthin Accumulation of H. pluvialis in Angled TL-PSBRs

**Figure 1:**
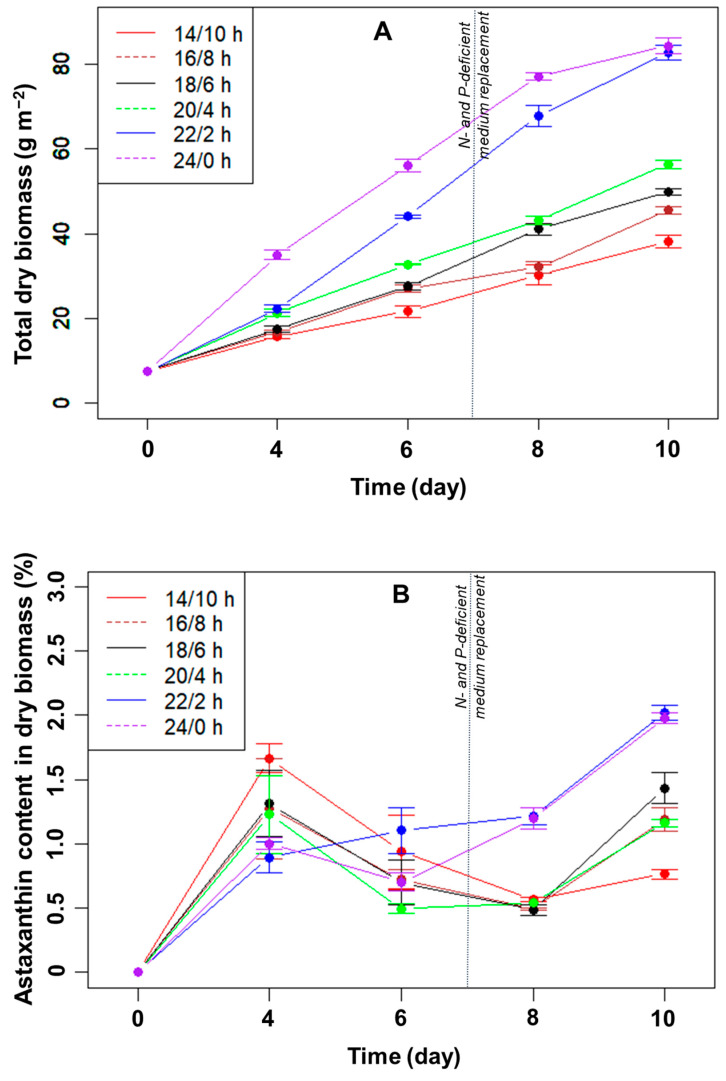
Total dry biomass (**A**) and astaxanthin (**B**) content in the dry biomass of *H. pluvialis* (in %) over time cultured in angled TL-PSBRs at different light/dark cycles.

Effect of the L/D cycle on the growth of *H. pluvialis*: Generally, the longer the light period in each light cycle, the higher the dry biomass of microalgae collected (Figure 1A). In all light/dark cycles, the total dry algal biomass in the biofilm increased linearly from day 4 to day 8. In the linear stage, the average growth rates of microalgae were highest when illuminated for 22 h and 24 h, reaching approximately 11.4 and 10.5 g m^−2^ day^−1^, respectively (*p* > 0.05, n = 3). The growth rates were only 3.6–5.9 g m^−2^ day^−1^ in the remaining light/dark cycles. After ten days, the total dry biomass was highest in the light/dark cycles of 22/2 h (82.8 g m^−2^) and 24/0 h (84.3 g m^−2^). The difference between these two light/dark cycles was not statistically significant at day 10 (*p* > 0.05, n = 3), but the total biomass was significantly higher than that of the other four cycles (*p* < 0.05, n = 3).

The biomass growth rate is often reduced when the thickness of the biofilm layer increases due to the formation of light and nutrient gradients inside the biofilm [5]. The cells in the biofilm no longer receive enough light and nutrients needed for growth. If the biofilm is approximately 350 μm thick, approximately 95% of the light is absorbed [17]. As a result, the cells near the bottom of the biofilm do not receive enough light for photosynthesis.

With the light–dark cycle of 14/10 h, the total dry biomass of microalgae was lowest, at 38.1 g m^−2^, after ten days of cultivation. In the light–dark cycles of 14/10 h, 16/8 h, 18/6 h, and 20/4 h, the biomass productivities were in the range of 3.1–4.9 g m^−2^ day^−1^. Meanwhile, the dry biomass productivities for ten days were 7.5 and 7.7 g m^−2^ day^−1^, respectively, in the light/dark cycles of 22/2 h and 24/0 h (*p* > 0.05, n = 3).

Effect of the L/D cycle on astaxanthin accumulation of *H. pluvialis*: The percentages of astaxanthin accumulated in the dry biomass over the cultivation period are shown in Figure 1B. After four days of immobilization, the astaxanthin content increased in all light/dark cycles, and the higher the total dry biomass, the lower the astaxanthin percentage. As the biofilm thickness increased, the percentage of cells in the green stage (with low astaxanthin content) in the total biomass increased, thus reducing the overall astaxanthin content. Consequently, on day four, the astaxanthin content was the highest (~1.7%) at a 14:10 h L/D cycle when the total biomass was the lowest (Figure 1A,B). From day 4 to 8, the percentage of astaxanthin remained relatively stable, at approximately 0.5–1%, presumably reflecting the stable exposed surface layer of the biofilm. At day 8, the astaxanthin content was higher (~1.2%) in those two biofilms with the most significant biomass increase (with 22 or 24 hrs light/day; Figure 1B), probably indicating that the nutrients may already have become limiting. After day 8, under N- and P-limitation, the astaxanthin content increased significantly for all L/D cycles (Figure 1B) and reached ~2% at the L/D cycles of 22:2 and 24:0 at day 10, significantly more than in the other L/D cycles (*p* < 0.05, n = 3).

**Figure 2:**
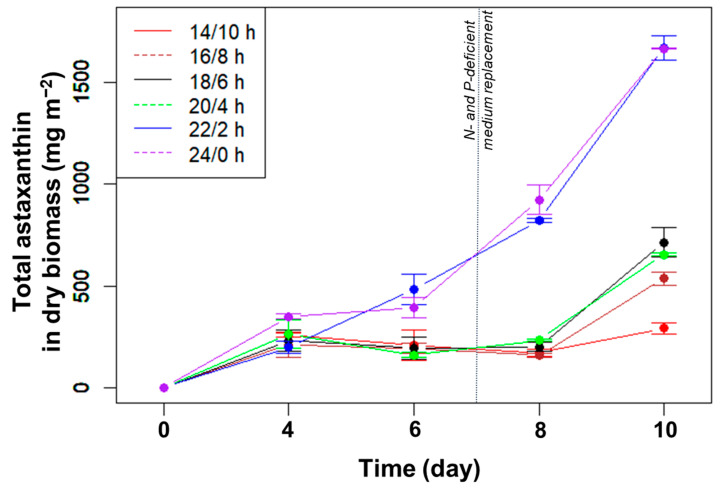
Total astaxanthin (mg m^−2^) accumulated in algal biomass over time cultured in angled TL-PSBRs at different light/dark cycles.

The total amount of astaxanthin accumulated in the biomass did not change significantly from day 4 to 6 (Figure 2). From day 6 to 8, at photoperiods with a light duration from 14 to 20 h, the total astaxanthin content remained unchanged despite the increased biomass due to the decreased percentage of astaxanthin. At these L/D cycles, the amount of astaxanthin only increased significantly in the last few days, i.e., under nutrient limitation. For the two longest light durations (22 and 24 h/day), the total astaxanthin increased rapidly from day 6 to 10 and reached the highest levels at day 10, with 1670 mg m^−2^ (*p* > 0.05, n = 3).

### 3.2. Effect of Carbon Source Supplementation on Growth and Astaxanthin Accumulation of H. pluvialis in Angled TL-PSBRs

#### 3.2.1. Effect of the Amount of CO_2_ Added in the Medium on *H. pluvialis* in Angled TL-PSBRs

**Figure 3:**
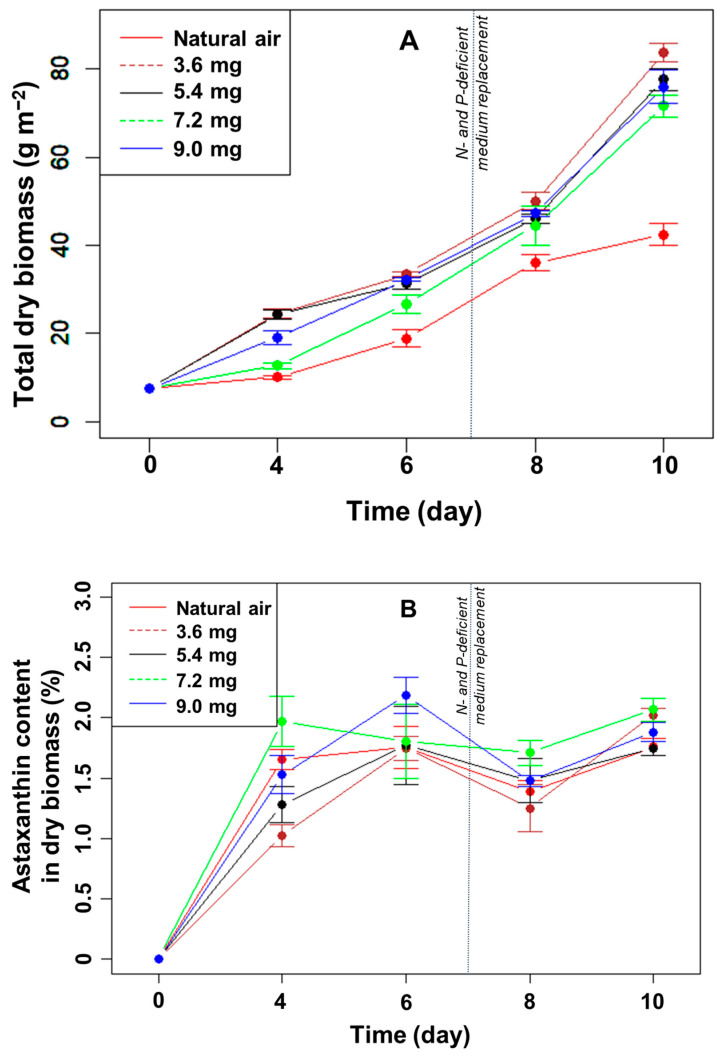
The change in the total dry biomass (**A**) and percentage of astaxanthin in the biomass (**B**) of *H. pluvialis* at different CO_2_ supplementation levels.

Effect of CO_2_ supplementation on algal biofilm growth: The total microalgal biomass in all treatments increased gradually over time (Figure 3A). After ten days of cultivation, the increase in biomass in the treatments with CO_2_ added to the medium was significantly higher than that of only filtered air; the difference was statistically significant (*p* < 0.05, n = 3). However, the increase in algal biomass was not linear with the increase in CO_2_ flowing into the system. The algal biomass in the biofilms after ten days reached 83.9, 77.7, 71.7, and 76.0 g m^−2^ with a supplemental CO_2_ flow of 3.6, 5.4, 7.2, and 9.0 mg min^−1^ (*p* > 0.05, n = 3), respectively.

A comparison between the control and the remaining treatments showed that the addition of CO_2_ to the medium greatly influenced the growth of algae in the biofilm during the culture period, in particular during nutrient limitation. Dissolved CO_2_ provides a vital carbon source for algal photosynthesis. However, adding a higher amount than 3.6 mg min^−1^ did not increase the algal biomass further.

Effect of CO_2_ supplementation on astaxanthin accumulation of *H. pluvialis* in biofilm: After ten days of culture, the accumulated astaxanthin content in the algal biomass, in %, did not differ significantly between the treatments with and without CO_2_ supplementation (*p* > 0.05, n = 3) (Figure 3B). The highest percentage of astaxanthin in the dry biomass was 2.1% in the treatment with CO_2_ addition at a flow rate of 7.2 mg min^−1^ into the culture medium. The lowest astaxanthin content was 1.8%, with a flow rate of CO_2_ of 5.4 mg min^−1^. Over the last days, when the N- and P-deficient media were replaced in the angled TL-PSBR system, the percentage of astaxanthin increased significantly at day ten in most of the CO_2_ supplements.

**Figure 4:**
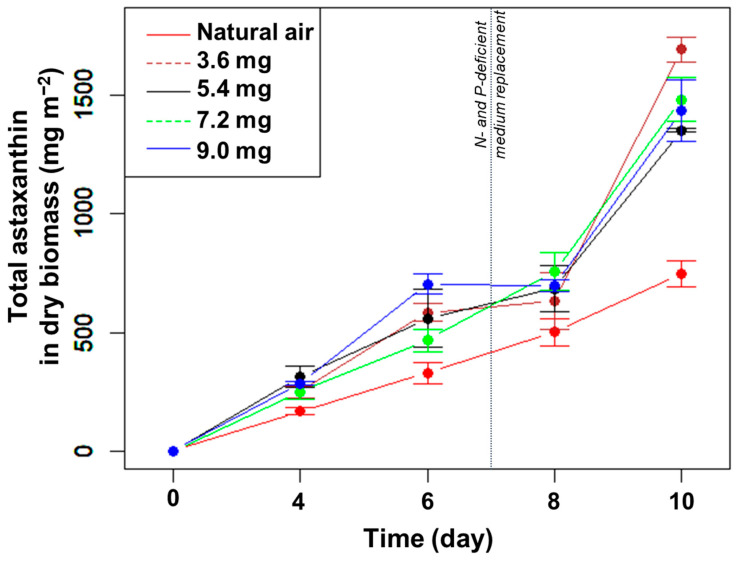
The change in total astaxanthin content in *H. pluvialis* at different CO_2_ supplementation levels.

The total amount of astaxanthin (mg m^−2^) increased gradually until day 8, except for the highest CO_2_ concentration between days 6 and 8 (Figure 4). Concomitant to the strong increase in biomass in the presence of added CO_2_ under N- and P-limitation (Figure 3A), the amount of astaxanthin also increased significantly between days 8 and 10 in the presence of added CO_2_ (but not to the same extent as in the control) (Figure 4). At day 10, the highest total amount of astaxanthin was 1693 mg m^−2^ in the CO_2_ supplement of 3.6 mg min^−1^, much higher than the control (*p* < 0.05, n = 3) but not significantly different compared to the other treatments (*p* > 0.05, n = 3) (Figure 4). Thus, adding CO_2_ did not directly affect the percentage of astaxanthin in the dry biomass but increased the total amount of astaxanthin per growth area under N- and P-limitation.

#### 3.2.2. Effect of the Concentration of NaHCO_3_ Dissolved in the Medium on *H. pluvialis* in Angled TL-PSBRs

**Figure 5:**
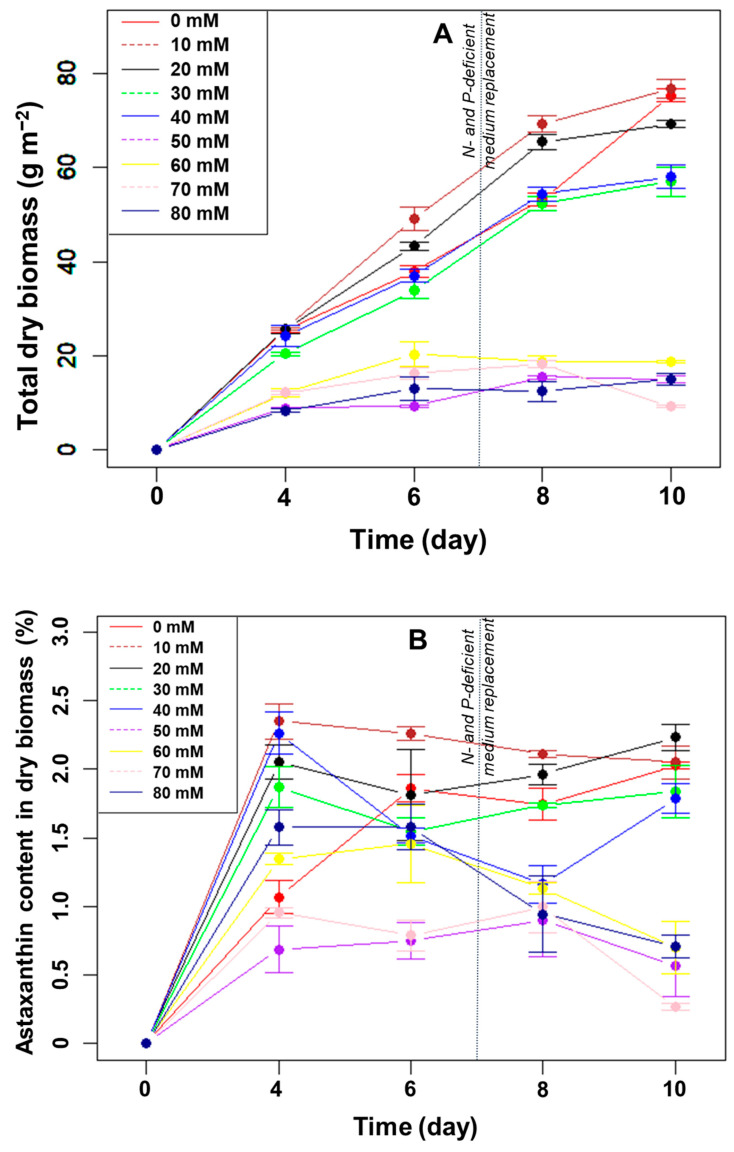
The change in total dry biomass (**A**) and percentage of astaxanthin in the biomass (**B**) of *H. pluvialis* at different amounts of NaHCO_3_ addition.

Effect of NaHCO_3_ supplementation on algal biofilm growth. The results of the effect of the concentrations of NaHCO_3_ on the growth of *H. pluvialis* in angled TL-PSBRs are presented in Figure 5A.

The results showed that *H. pluvialis* continued to grow and increased in biomass when cultured in a medium supplemented with 10–40 mM NaHCO_3_. When the concentration of NaHCO_3_ exceeded 40 mM, the growth of microalgae was almost negligible, indicating that the high concentration of NaHCO_3_ inhibited the growth of the microalgae. The total dry biomass was very low, at 9.2–18.8 g m^−2^, when adding NaHCO_3_ with a concentration of 50–80 mM.

For the addition of NaHCO_3_ from 10–40 mM, the dry biomass gradually increased from day 0 to day 8. At day 8, the total dry biomass was significantly higher at the two lower NaHCO_3_ concentrations (10 and 20 mM) than in either the control or at the two higher NaHCO_3_ concentrations (30 and 40 mM) (Figure 5A). Under N- and P-depletion (days 8–10), the biomass in the control increased significantly, while the increase was much slower with the four NaHCO_3_ concentrations. Therefore, the total dry biomass after ten days of cultivation was highest in the control and at the lowest NaHCO_3_ concentration but significantly higher than at all other NaHCO_3_ concentrations (Figure 5A).

Thus, adding NaHCO_3_ over ten days of culture was ineffective at increasing the biomass compared to adding CO_2_. In addition, the algae grew favorably at the investigated concentrations, when the NaHCO_3_ concentration was less than or equal to 20 mM in the BG11 H medium.

Effect of NaHCO_3_ supplementation on the astaxanthin accumulation of *H. pluvialis* in the biofilm: After ten days of cultivation, the accumulated astaxanthin content, in %, of the dry biomass of algae was significantly different (*p* < 0.05, n = 3) between the groups supplemented with NaHCO_3_ concentrations above 40 mM and those with less than 40 mM (Figure 5B). The highest percentage of astaxanthin in the dry biomass was 2.2% when 20 mM NaHCO_3_ was added to the culture medium. In the control and at the concentrations of NaHCO_3_ of 10 mM, 30 mM, and 40 mM, the percentages of astaxanthin in the dry biomass were 2.1%, 2.0%, 1.8%, and 1.8%, respectively. However, the differences were not statistically significant (*p* > 0.05, n = 3). Meanwhile, the percentages of astaxanthin were less than 1.0% in the cases of NaHCO_3_ supplementation above 40 mM.

Similar to the CO_2_ carbon source experiment, the values of dry biomass and astaxanthin content did not increase linearly with the increase in dissolved NaHCO_3_ in the medium. Remarkably, however, the results recorded on day 4 of cultivation indicated that when the medium was supplemented with NaHCO_3_ with a concentration of 10–30 mM, the astaxanthin content in the dry biomass was significantly higher than that of the control (*p* < 0.05, n = 3). From day 6 to 8, the percentage of astaxanthin in the dry biomass remained constant or decreased at most NaHCO_3_ concentrations.

**Figure 6:**
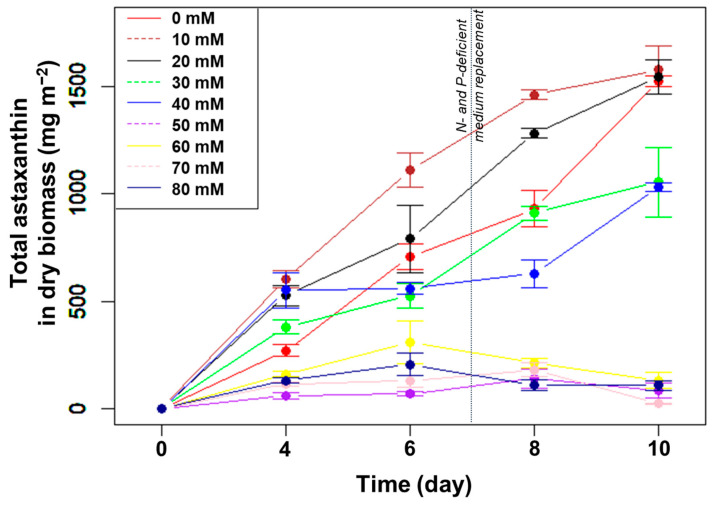
The change in astaxanthin content of *H. pluvialis*, in m^−2^, cultured in angled TL-PSBRs with different amounts of NaHCO_3_ addition.

The total astaxanthin amount, in m^−2^, increased gradually at NaHCO_3_ concentrations from 0, 10, 20, 30 to 40 mM. Meanwhile, the amount of astaxanthin remained low at the higher (>40 mM) NaHCO_3_ concentrations (Figure 6). After ten days of culture, the total amount astaxanthin obtained was highest in the control and the two lowest (10 and 20 mM) NaHCO_3_ concentrations, reaching 1527, 1578, and 1547 mg m^−2^, respectively (*p* > 0.05, n = 3).

After ten days of cultivation, microscopic observations of algal cells from the experiments with 50–80 mM NaHCO_3_ supplementations revealed many dead cells in addition to akinetes (results not shown), possibly explaining the low biomass obtained at these bicarbonate concentrations.

## 4. Discussion

Our initial study using LED light to cultivate *H. pluvialis* in an angled TL-PSBR at the pilot scale resulted in relatively low amounts of astaxanthin on the biofilm when applying a light/dark cycle of 14/10 h compared to illumination with high-pressure sodium lamps [6,13,24]. However, increasing the lighting time per day with an LED effectively increased the algal biomass and the amount of astaxanthin obtained, as shown in this study. This helps to shorten the cultivation time and, thus, reduces the risk of contamination of the biofilm in the angled TL-PSBRs. Continuous lighting can induce oxidative stress and initiate astaxanthin accumulation, which is further enhanced by nutrient limitation in suspension cultures [16]. In immobilized cultivation, the highest total astaxanthin content was also obtained at the light/dark cycle of 22/2 h or in continuous light despite the difference of the culture system compared to suspension cultures.

**Figure 7:**
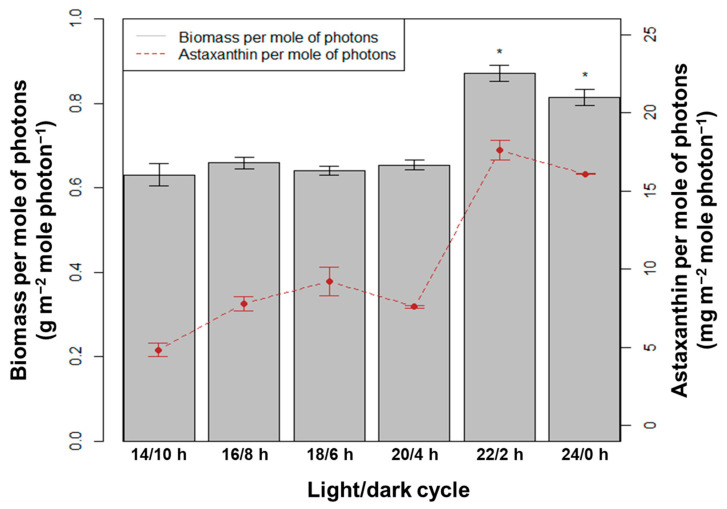
Biomass and astaxanthin in *H. pluvialis* per mole of photons at different light/dark cycles; the asterisks indicate statistically significant differences.

With the average blue + red LED light intensity used in the system of 120 µmol photons m^−2^ s^−1^, the total number of moles of photons was calculated for the 10-day culture period. The dry biomass and amount of astaxanthin per mole of photons used were determined (Figure 7). Statistical analysis showed that the efficiency of the dry biomass and astaxanthin accumulation was highest in the light/dark cycles of 22/2 h and 24/0 h, compared to the other L/D cycles (*p* < 0.05, n = 3). With the 22/2 h, the dry biomass and astaxanthin yield reached 0.87 g m^−2^ mole photon^−1^ and 17.6 mg m^−2^ mole photon^−1^, respectively. These results are equivalent to the cycle of 24/0 h, and the values were, respectively, 0.81 g m^−2^ mole photon^−1^ and 16.08 mg m^−2^ mole photon^−1^ (*p* > 0.05, n = 3). Therefore, increasing the lighting time per day to 22 h is the threshold value of saturation. LED light with a longer exposure time still resulted in significantly higher biomass and astaxanthin productivities per mole photons compared to high-pressure sodium light with a shorter light/dark cycle [6,12]. This explains why the production costs of astaxanthin when using LED light sources are always lower than with other artificial light sources [15,25,26,27].

In suspension cultivation, the addition of NaHCO_3_ induced phase transition and astaxanthin accumulation of *H. pluvialis* [23,28]. The addition of 100 mM bicarbonate in combination with an N- and P-deficient medium and a high light intensity showed an inhibitory effect on algal growth; the cell density decreased, but the percentage of astaxanthin was up to 4.9% of dry biomass after only three days [29]. However, if the suspension period was longer, bicarbonate concentrations above 80 mM showed inhibition of astaxanthin accumulation after 18 days [28]. The percentage of astaxanthin was highest, at ~3% of dry biomass, when the bicarbonate concentration was at 20 mM [23]. Despite the different cultivation systems, the results of the immobilization of *H. pluvialis* in angled TL-PSBRs also showed that astaxanthin accumulation was favored at low NaHCO_3_ concentrations in replete culture medium. In biofilms, lower bicarbonate concentrations can be used over a longer period of culture to increase the total dry biomass and astaxanthin amount. Furthermore, bicarbonate may contribute to an increase in the C/N ratio under N- and P-depletion, and algal cells still photosynthesize, accumulate carotenes (including astaxanthin), and increase in cell size. Therefore, algal biomass increased during this period, although the number of cells may not change.

High bicarbonate concentrations are only effective in inducing cells to accumulate astaxanthin for a short time but cause cell death and decrease cell density over longer cultivation times. NaHCO_3_ makes the environment alkaline and exceeds the favorable pH threshold for *Haematococcus* growth (6.5–8) [30]. At 10 and 20 mM NaHCO_3_ concentrations, adding 3.6 mg min^−1^ CO_2_ helped to maintain the pH in the optimal range for the growth of *H. pluvialis* during the immobilization in biofilms. With higher concentrations of NaHCO_3_, the addition of CO_2_ was not sufficient to reduce the pH to a range favorable for microalgal growth.

Bicarbonate is an inorganic carbon source with the advantage of high solubility in the culture medium due to the fact of its ionic form. *H. pluvialis* cells can utilize this carbon source by activating carbonic anhydrase, which converts HCO_3_^−^ to CO_2_ [31]. However, the absorption of bicarbonate ions across cell membranes and chloroplasts requires transport proteins and energy, while CO_2_ can diffuse freely [32]. In suspension, the algal cells can receive a uniform carbon addition through stirring or aeration. For immobilized cultivation, the process of transferring CO_2_ or HCO_3_^−^ from the BG11-H medium to the cells in the biofilms is based on passive diffusion and capillary conduction [5]. In an angled TL-PSBR, when the culture medium containing inorganic carbon is pumped to the source layer, the carbon sources follow the capillary flow to the substrate layer and diffuse to the algal layers in the biofilms. The addition of CO_2_ showed high biomass growth efficiency, but its low solubility along with the prolonged diffusion rate in water (10^4^ times slower than in air) makes the increase in the amount of CO_2_ supplement ineffective [33]. In other words, the ability to add CO_2_ to the nutrient medium used in the TL-PSBRs is limited.

Studies on immobilized *H. pluvialis* cultivation in small-scale TL-PSBRs have yielded between 3.7 and 19.4 g m^−2^ day^−1^ dry biomass and an astaxanthin content of microalgae of approximately 1.3–3.5% after 7 or 16 days when using high-pressure sodium light sources and adding CO_2_ to the gas phase [6,7,24]. In our study, with the use of CO_2_ and low concentrations of NaHCO_3_, the maximum growth rate obtained was 11.4 g m^−2^ day^−1^. This growth rate is higher than in almost all studies performed in suspension systems (values converted to “per surface area”) [8]. Compared with the same inclined TL-PSBR system, the growth rate is equivalent to using light from a high-pressure sodium lamp [8,12]. However, the increase in biomass was still significantly lower than that of *H. pluvialis* cultivation in a vertical small-scale TL-PSBR. Kiperstok et al. [6] reported a maximum of 19.4 g m^−2^ day^−1^ productivity in vertical TL-PSBRs, and the astaxanthin content in the biomass reached 1.5% at a high light intensity of 1015 μmol photons m^−2^ s^−1^. The LED light intensity of 120 μmol photons m^−2^ s^−1^ is probably limiting the astaxanthin accumulation, as well as biomass productivity. Furthermore, the results show that the addition of CO_2_ to the gas phase in the TL-PSBR has an important role for the photosynthesis of microalgae. However, the gas phase addition of CO_2_ is only feasible with systems of a small size and low air circulation.

The low LED light intensity may limit the ratio of astaxanthin in dry biomass. Initially, our experiments using LEDs in large-scale angled TL-PSBRs with a 14/10 h light/dark cycle showed a biomass productivity of 3.3 g m^−2^ day^−1^ and an astaxanthin content of 1.3% in the dry biomass [14]. In this study, with the light cycle 22/2 h and the addition of inorganic carbon sources to the medium in the same TL-PSBRs, the average biomass productivity was approximately 7.5 g m^−2^ day^−1^, and the astaxanthin content was approximately 2.0% after ten days. In further studies, the accumulation of astaxanthin can be improved if other factors that promote astaxanthin accumulation are adjusted at the end of the culture period in TL-PSBRs. Recently, a green algal strain of *Chromochloris zofingiensis* showed a yield of astaxanthin 5.4 times higher than that of *H. pluvialis* [10,34]. Therefore, the cultivation of high-productivity algal strains in TL-PSBR should be conducted in future studies.

## 5. Conclusions

Increasing the light duration of artificial illumination in each L/D cycle with blue and red LED light sources stimulated growth and astaxanthin accumulation of *H. pluvialis* immobilized in pilot-scale angled TL-PSBRs. The supplementation of 10–20 mM NaHCO_3_ over ten days of *H. pluvialis* cultivation increased the total astaxanthin accumulation, in m^−2^, growth area compared to CO_2_ alone in a replete culture medium. A concentration of NaHCO_3_ higher than 30 mM reduced the total astaxanthin productivity. The addition of CO_2_ to the BG11-H medium was limited by its solubility in water. Adding more than 3.6 mg min^−1^ did not increase growth or biomass accumulation.

## Data Availability

All data generated or analyzed during this study are included in this published article.

## References

[1] Podola B., Li T., Melkonian M. (2017). Porous Substrate Bioreactors: A Paradigm Shift in Microalgal Biotechnology?. Trends Biotechnol..

[2] Zhuang L.-L., Yu D., Zhang J., Liu F., Wu Y.-H., Zhang T.-Y., Dao G.-H., Hu H.-Y. (2018). The Characteristics and Influencing Factors of the Attached Microalgae Cultivation: A Review. Renew. Sustain. Energy Rev..

[3] Pierobon S.C., Cheng X., Graham P.J., Nguyen B., Karakolis E.G., Sinton D. (2017). Emerging Microalgae Technology: A Review. Sustain. Energy Fuels.

[4] Benstein R.M., Cebi Z., Podola B., Melkonian M. (2014). Immobilized Growth of the Peridinin-Producing Marine Dinoflagellate *Symbiodinium* in a Simple Biofilm Photobioreactor. Mar. Biotechnol..

[5] Li T., Strous M., Melkonian M. (2017). Biofilm-Based Photobioreactors: Their Design and Improving Productivity through Efficient Supply of Dissolved Inorganic Carbon. FEMS Microbiol. Lett..

[6] Kiperstok A.C., Sebestyén P., Podola B., Melkonian M. (2017). Biofilm Cultivation of *Haematococcus pluvialis* Enables a Highly Productive One-Phase Process for Astaxanthin Production Using High Light Intensities. Algal. Res..

[7] Zhang W., Wang J., Wang J., Liu T. (2014). Attached Cultivation of *Haematococcus pluvialis* for Astaxanthin Production. Bioresour. Technol..

[8] Tran H.D., Do T.T., Le T.L., Tran-Nguyen M.L., Pham C.H., Melkonian M. (2019). Cultivation of *Haematococcus pluvialis* for Astaxanthin Production on Angled Bench-Scale and Large-Scale Biofilm-Based Photobioreactors. Vietnam J. Sci. Technol. Eng..

[9] Bas T.G., Contreras A., Oliu C.A., Abarca A., Bas T.G., Contreras A., Oliu C.A., Abarca A. (2021). Determinants of Astaxanthin Industrial-Scale Production under Stress Caused by Light Photoperiod Management of *Haematococcus pluvialis* Cultivation. Lat. Am. J. Aquat. Res..

[10] Oslan S.N.H., Shoparwe N.F., Yusoff A.H., Rahim A.A., Chang C.S., Tan J.S., Oslan S.N., Arumugam K., Bin Ariff A., Sulaiman A.Z. (2021). A Review on *Haematococcus pluvialis* Bioprocess Optimization of Green and Red Stage Culture Conditions for the Production of Natural Astaxanthin. Biomolecules.

[11] Pereira S., Otero A. (2020). *Haematococcus pluvialis* Bioprocess Optimization: Effect of Light Quality, Temperature and Irradiance on Growth, Pigment Content and Photosynthetic Response. Algal Res..

[12] Do T.T., Ong B.N., Nguyen Tran M.L., Nguyen D., Melkonian M., Tran H.D. (2019). Biomass and Astaxanthin Productivities of *Haematococcus pluvialis* in an Angled Twin-Layer Porous Substrate Photobioreactor: Effect of Inoculum Density and Storage Time. Biology.

[13] Do T.-T., Tran-Thi B.-H., Ong B.-N., Le T.-L., Nguyen T.-C., Quan Q.-D., Le T.-C., Tran D.-L., Melkonian M., Tran H.-D. (2021). Effects of Red and Blue Light Emitting Diodes on Biomass and Astaxanthin of *Haematococcus pluvialis* in Pilot Scaleangled Twin-Layer Porous Substrate Photobioreactors. Vietnam J. Sci. Technol. Eng..

[14] Do T.-T., Ong B.-N., Le T.-L., Nguyen T.-C., Tran-Thi B.-H., Thu Hien B.T., Melkonian M., Tran H.-D. (2021). Growth of *Haematococcus pluvialis* on a Small-Scale Angled Porous Substrate Photobioreactor for Green Stage Biomass. Appl. Sci..

[15] Wong Y. (2016). Effects of Light Intensity, Illumination Cycles on Microalgae *Haematococcus pluvialis* for Production of Astaxanthin. J. Mar. Biol. Aquac..

[16] Domínguez A., Pereira S., Otero A. (2019). Does *Haematococcus pluvialis* Need to Sleep?. Algal Res..

[17] Li T., Podola B., Melkonian M. (2016). Investigating Dynamic Processes in a Porous Substrate Biofilm Photobioreactor—A Modeling Approach. Algal Res..

[18] Ji C., Wang J., Li R., Liu T. (2017). Modeling of Carbon Dioxide Mass Transfer Behavior in Attached Cultivation Photobioreactor Using the Analysis of the PH Profiles. Bioprocess Biosyst. Eng..

[19] Pham K.T., Nguyen T.C., Luong T.H., Dang P.H., Vu D.C., Do T.N., Phi T.C.M., Nguyen D.B. (2018). Influence of Inoculum Size, CO^2^ Concentration and LEDs on the Growth of Green Microalgae *Haematococcus pluvialis* Flotow. Vietnam J. Sci. Technol..

[20] Schultze L.K.P., Simon M.-V., Li T., Langenbach D., Podola B., Melkonian M. (2015). High Light and Carbon Dioxide Optimize Surface Productivity in a Twin-Layer Biofilm Photobioreactor. Algal Res..

[21] Liyanaarachchi V.C., Premaratne M., Viraj Nimarshana P.H., Udayangani Ariyadasa T. Investigation of the Effect of Organic and Inorganic Carbon on Biomass Production and Astaxanthin Accumulation of the Microalga *Haematococcus pluvialis* Using Artificial Neural Network. Proceedings of the 2020 IEEE 17th India Council International Conference (INDICON).

[22] Devgoswami C., Kalita M., Talukdar J., Bora R., Sharma P. (2011). Studies on the Growth Behavior of Chlorella, *Haematococcus* and *Scenedesmus Sp*. in Culture Media with Different Concentrations of Sodium Bicarbonate and Carbon Dioxide Gas. Afr. J. Biotechnol..

[23] Kang C.D., Lee J.S., Park T.H., Sim S.J. (2005). Comparison of Heterotrophic and Photoautotrophic Induction on Astaxanthin Production by *Haematococcus pluvialis*. Appl. Microbiol. Biotechnol..

[24] Wan M., Hou D., Li Y., Fan J., Huang J., Liang S., Wang W., Pan R., Wang J., Li S. (2014). The Effective Photoinduction of *Haematococcus pluvialis* for Accumulating Astaxanthin with Attached Cultivation. Bioresour. Technol..

[25] Al-Amshawee S., Mohd Yunus M.Y. (2020). Influence of Light Emitting Diode (LED) on Microalgae. J. Chem. Eng. Ind. Biotechnol..

[26] Cui S., Li X., Zhang Y., Wu X., He L. (2019). Design of Automatic Illumination Culture System for *Haematococcus pluvialis* Based on LED. Proceedings of the Chinese Intelligent Automation Conference.

[27] Ma R., Thomas-Hall S.R., Chua E.T., Eltanahy E., Netzel M.E., Netzel G., Lu Y., Schenk P.M. (2018). Blue Light Enhances Astaxanthin Biosynthesis Metabolism and Extraction Efficiency in *Haematococcus pluvialis* by Inducing Haematocyst Germination. Algal Res..

[28] Luu T.T., Hoang T.-L.A., Ngo T.-H.T., Hoang T.-M.H., Dang D.H. (2014). Effect of Bicarbonate Concentration on Astaxanthin Accumulation of Green Microalga of Haematococcus pluvialis. Proceedings of the 3rd Academic Conference on Natural Science for Master and PhD Students from Asean Countries.

[29] Luu T.T. (2017). Study on Biological Characteristics and Astaxanthin Rich Biomass Production of Microalga Haematococcus pluvialis Flotow to Applications for Aquaculture.

[30] Sarada R., Tripathi U., Ravishankar G.A. (2002). Influence of Stress on Astaxanthin Production in *Haematococcus pluvialis* Grown under Different Culture Conditions. Process Biochem..

[31] Gu W., Li H., Zhao P., Yu R., Pan G., Gao S., Xie X., Huang A., He L., Wang G. (2014). Quantitative Proteomic Analysis of Thylakoid from Two Microalgae (*Haematococcus pluvialis* and *Dunaliella salina*) Reveals Two Different High Light-Responsive Strategies. Sci. Rep..

[32] Kaplan A., Reinhold L. (1999). CO^2^ Concentrating Mechanisms in Photosynthetic Microorganisms. Annu. Rev. Plant Biol..

[33] Thorbjørn A., Moldrup P., Blendstrup H., Komatsu T., Rolston D.E. (2008). A Gas Diffusivity Model Based on Air-, Solid-, and Water-Phase Resistance in Variably Saturated Soil. Vadose Zone J..

[34] Chen Q., Chen Y., Xu Q., Jin H., Hu Q., Han D. (2022). Effective Two-Stage Heterotrophic Cultivation of the Unicellular Green Microalga Chromochloris Zofingiensis Enabled Ultrahigh Biomass and Astaxanthin Production. Front. Bioeng. Biotechnol..

